# White Matter-Gray Matter Correlation Analysis Based on White Matter Functional Gradient †

**DOI:** 10.3390/brainsci15010026

**Published:** 2024-12-29

**Authors:** Zhengjie Li, Jiajun Liu, Jianhui Zheng, Luying Li, Ying Fu, Zhipeng Yang

**Affiliations:** 1 College of Electronic Engineering, Chengdu University of Information Technology, Chengdu 610225, China; lizhengjie126@163.com (Z.L.); jiajliu0701@163.com (J.L.); zjhworkemail@163.com (J.Z.); fuying@cuit.edu.cn (Y.F.); 2 Department of Radiology, Huaxi MR Research Center, West China Hospital, Sichuan University, Chengdu 610017, China; liluying126@163.com

**Keywords:** WM, rs-fMRI, functional gradient, CC, asymmetry

## Abstract

Background: The spontaneous fluctuations in functional magnetic resonance imaging (fMRI) signals of the brain’s gray matter (GM) have been interpreted as representations of neural activity variations. In previous research, white matter (WM) signals, often considered noise, have also been demonstrated to reflect characteristics of functional activity and interactions among different brain regions. Recently, functional gradients have gained significant attention due to their success in characterizing the functional organization of the whole brain. However, previous studies on brain functional gradients have predominantly focused on GM, neglecting valuable functional information within WM. Methods: In this paper, we have elucidated the symmetrical nature of the functional hierarchy in the left and right brain hemispheres in healthy individuals, utilizing the principal functional gradient of the whole-brain WM while also accounting for gender differences. Results: Interestingly, both males and females exhibit a similar degree of asymmetry in their brain regions, albeit with distinct regional variations. Additionally, we have thoroughly examined and analyzed the distribution of functional gradient values in the spatial structure of the corpus callosum (CC) independently, revealing that a simple one-to-one correspondence between structure and function is absent. This phenomenon may be associated with the intricacy of their internal structural connectivity. Conclusions: We suggest that the functional gradients within the WM regions offer a fresh perspective for investigating the structural and functional characteristics of WM and may provide insights into the regulation of neural activity between GM and WM.

## 1. Introduction

Functional magnetic resonance imaging (fMRI) has been extensively employed in research on neuroscience and cognitive science, with its main principle being the detection of neural activity in the gray matter (GM) of the brain through changes in blood oxygen level-dependent (BOLD) effects. However, the existence and validity of BOLD signals in the white matter (WM) of the brain remain contentious issues. Previous studies have found that when under stimulation, BOLD signals in the corpus callosum (CC) are activated simultaneously with the functional areas of the GM. Furthermore, changes in BOLD signals have also been observed in related WM structures under specific functional stimuli [1,2,3]. Recently, a growing body of studies has shown that BOLD signals in WM reflect neural activity in the brain [4,5,6,7,8]. For instance, during the resting state, the functional signal in the white matter is highly homogeneous and effectively describes neuronal activity [9]. Peer et al. demonstrated the existence of 12 symmetric WM functional networks (WM-FNs) by performing K-means clustering of WM voxel resting-state fMRI (rs-fMRI) data, similar to the functional organization observed in the GM, suggesting that WM also acts as a network of functional modules which interact with each other [10]. Moreover, WM has been shown to play an active and significant role in functional brain coupling during the resting state [11]. Huang et al. used independent component analysis (ICA) to analyze the WM fMRI signal and identified functional signal changes and functional structures in WM [12]. Additionally, Li et al. found that WM BOLD signals may exhibit different time courses during the resting state, potentially reflecting changes in anatomical, neurovascular, and functional coupling in different voxel populations [5]. The WM BOLD signal reflecting the brain’s neural activity has also been confirmed in several studies on psychiatric disorders. For example, patients with schizophrenia exhibit abnormalities in the WM perceptual-motor functional network [13], and patients with Parkinson’s disease demonstrate distinct small-world characteristics in their WM functional networks compared with healthy individuals [14]. These studies provide evidence for the presence of functional brain activity in WM.

The WM region of the brain, positioned beneath the cortical GM, is composed of neuronal fibers enveloped by myelin, an insulating sheath which facilitates electrical conduction [15]. Previous investigations have effectively utilized diffusion tensor imaging (DTI) to elucidate the intricate architecture of WM. Despite its critical involvement in brain activity, current research methods have predominantly focused on the structural aspect of WM, with limited consideration of its functional organization.

Functioning as a relay which regulates the distribution of action potentials and coordinates inter-regional communication in the brain, WM has been shown to be instrumental in underlying seizure dynamics [16]. For instance, epilepsy is more common in patients with abnormalities in the CC [17]. The above studies highlight the importance of WM functionality and underscore that specific WM regions can offer credible physiological or pathological explanations for certain neurological disorders.

Recent advancements in neuroimaging and brain neuroscience research have made significant strides in mapping spatial gradients to study functional structures. In the context of brain connectivity research, a gradient refers to the continuous variation in functional activity across different brain regions, often represented along a spectrum which highlights the varying degrees of interaction and processing between these regions. Specifically, functional gradients describe the transition from brain areas involved in basic sensory processing to those engaged in higher-order cognitive functions, reflecting a hierarchical pattern of neural activity [18]. Macroscopic gradients integrate system information into more abstract spatial representations and typically extend in a stepwise manner along adjacent cortical areas [19,20,21]. These gradients have been well characterized in different brain tissues up to now, including multi-scale brain structures such as the cerebral cortex, cerebellum, hippocampus, thalamus, and temporal lobe [22,23,24]. Margulies et al. employed the diffusion map embedding algorithm to provide a concise and powerful description of the “functional gradient” of resting-state functional connectivity (FC) in the cerebral cortex [18]. Guell et al. demonstrated for the first time that cerebellar functional regions exhibit a progressive organization from primary to transmodal regions using the concept of functional gradients [24]. Vos de Wael et al. identified two main FC gradients using hippocampal–cortical connections, with the first gradient corresponding to long-axis anatomical subdivision and meta-analytic gradients while the second gradient reflects the internal microstructure [25]. Furthermore, functional gradients have also shown potential applications in psychiatric and neurodegenerative disorders, such as autism and epilepsy [26,27]. However, these studies have primarily focused on the GM regions of the brain, and exploration of functional gradients within WM areas remains underdeveloped.

Building on recent advances in functional gradient research, we employed resting-state fMRI (rs-fMRI) to investigate functional gradients in white matter (WM). The objective of this study is to enhance our understanding of the macroscopic structure–function relationship within white matter through the analytical examination of WM functional gradients. Specifically, we aimed to extract functional gradients from white matter-gray matter functional connectivity (WM-GM FC), explore interhemispheric asymmetry across different groups, and examine the relationship between structure and function within specific white matter organizations. We focused on the first three gradients and examined four key issues: (1) functional gradients mapping back to brain WM structures; (2) the asymmetry of the brain’s WM functional gradients; (3) the distribution of the functional gradient values in the CC structure; and (4) the functional connectivity between WM and GM, as reflected by the functional gradient of WM.

This article is a revised and expanded version of a paper entitled “Human Brain White Matter Function Analysis Based on Functional Gradient”, which was presented at the 2022 4th International Conference on Frontiers Technology of Information and Computer (ICFTIC) [28].

## 2. Materials and Methods

### 2.1. Datasets and Preprocessing

The data for normal subjects used in this work were obtained from the Human Connectome Project (HCP) S1200 release, which consists of behavioral and 3T MRI data from 1206 healthy young adults (aged 22–35 years with a gender distribution of 45% male and 55% female) collected from 2012 to 2015 [29] with the following parameters: repetition time (TR) = 720 ms, temporal echo (TE) = 33.1 ms, flip angle (FA) = 52°, resolution = 2.0 mm, and matrix = 104 × 90. In this work, we mainly used rs-fMRI data and T1-weighted MRI from 100 randomly selected subjects (50 males and 50 females). Preprocessed data from the HCP minimal preprocessing pipeline were used, which included gradient distortion correction, head motion correction, image distortion correction, spatial normalization to the Montreal Neurological Institute (MNI) standard, and intensity normalization [30,31]. In addition to these steps, we regressed the head motion and CSF signals, applied bandpass filters (0.01–0.1 Hz) to reduce noise, and performed spatial smoothing transformation, where the smoothing work was provided by the DPABI toolbox [32].

### 2.2. Creation of Group-Level WM and GM Masks

To obtain a group-level WM mask, we employed the T1 structural data of each subject, which were segmented using the SPM segmentation algorithm to minimize the impact of the partial differential equation (PDE). Specifically, for each subject, we categorized each voxel into three classes—white matter, gray matter, and cerebrospinal fluid—based on the highest probability from the three segmented images. This step generated binary WM and GM masks for each subject. Subsequently, the two masks were averaged across all subjects. Finally, the group-level WM mask was derived by applying a threshold of 0.95 to the averaged primary WM mask. More specifically, the voxels of the resulting mask were selected and identified as WM to ensure the validity of the data if they exceeded 95% of all subjects [33]. Among them, we used the spatial location to obtain a total of 3780 voxels in the GM as a new GM mask. We obtained 48 WM bundles from the JHU-ICBM-labels-2 mm WM atlas [34]. To match the group-level WM template, we utilized the group-level WM mask to constrain the size of the CC.

### 2.3. Calculation of WM Functional Gradients Based on WM-GM Connectome

We applied the BrainSpace toolbox to estimate the functional gradient of each WM voxel for the normal subjects [35]. BrainSpace is a toolbox designed for analyzing brain connectomics data and identifying brain functional gradients. It is particularly well suited for extracting global structures and gradient information from similarity or connectivity data between brain regions. The typical workflow of BrainSpace is as follows.

Step 1: The affinity matrix is computed by applying the normalized angle function to the connectivity matrix. The normalized angle function is a method used to assess the similarity between brain regions. In this context, similarity refers to the degree of resemblance between brain regions in terms of their connectivity patterns. The affinity matrix, however, represents a transformation of this similarity into a structured matrix which encodes the strength of functional relationships across brain regions. Specifically, it first calculates the cosine similarity between the feature vectors of two regions, representing their directional similarity. Then, the inverse cosine function is applied to determine the angle between the two vectors. Finally, the similarity score is normalized to the range [0, 1] by dividing the angle by π and subtracting the result. This function is an extension of cosine similarity, and it constructs a similarity matrix for the subsequent gradient generation process.

**Figure 1:**
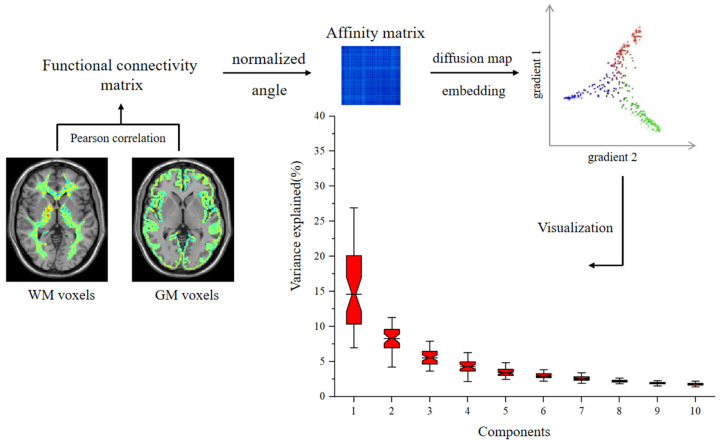
The WM FC matrix was transformed into an affinity matrix, and diffusion map embedding was used to calculate functional gradients based on the BrainSpace toolbox.

Step 2: The affinity matrix is then mapped into a lower-dimensional space using the diffusion map embedding algorithm, capturing the global structure and functional gradients of the data. The resulting scores are visualized to reveal the global structure and functional organization of brain networks. In diffusion map embedding, the diffusion matrix, which represents the diffusion probabilities between data points, is first computed. Then, eigenvalue decomposition is performed to extract the principal eigenvectors (or diffusion modes) of the diffusion matrix. These eigenvectors represent the primary directions of variation in the data and correspond to the key components of the functional gradients. Using these eigenvectors, the distribution of brain regions along the functional gradients is revealed, providing insights into how brain regions are organized and collaborate functionally. We plotted the spatial representation of WM functions based on WM-GM connectomes, using diffusion map embedding to capture the spatial similarity of the connection profiles between WM voxels in the normal subjects (Figure 1).

Firstly, the individual WM-GM FC matrix was constructed between the BOLD signal for each voxel in the WM and the BOLD signal for each voxel in the GM for each subject’s brain using Pearson’s correlation analysis. In total, for each subject, a correlation matrix was evaluated to characterize the WM-GM functional connectome. Secondly, we employed a kernel function called normalized angle, which captures the connectivity of each seed region, to construct the affinity matrix from each subject’s WM-GM functional connectome. Finally, we employed a diffusion map embedding algorithm to capture similarities between voxels and obtain multiple consecutive gradient components (i.e., functional gradients). These consecutive gradient components represent a sequence of functional gradients which capture the hierarchical and continuous transition in brain activity. They are derived by analyzing the variation in functional connectivity patterns across different brain regions, revealing the functional hierarchy from lower-level sensory processing to higher-order cognitive functions. These individual components from the functional gradient were then extracted and mapped to the WM space to visualize macroscopic changes in the overall connectivity pattern. The diffusion map embedding algorithm is known for its robustness to noise, making it a favorable choice compared with other nonlinear dimensionality reduction techniques [24].

The specific method flow is as follows: (1) the group level gradient template (generated from the average connection matrix based on all subjects) was estimated, and (2) Procrustes rotation was executed to align each subject to the template to obtain individual functional gradients. Following prior research, we retained the top 10% functional connections in each row to construct an affinity matrix [22,27]. This matrix effectively characterizes the similarity between voxels while preserving essential information. In addition, the diffusion map embedding algorithm is controlled by a parameter α [27]. In the diffusion map embedding algorithm, α is a key hyperparameter which controls the degree of anisotropic diffusion in the process. It influences how relationships between data points are propagated, thereby affecting the calculation of the gradients. In BrainSpace, the default value of α is typically set to 0.5, providing a balance between both global and local structures. We followed the previous suggestion and set α = 0.5 to preserve the global relationships between the data points in the embedding space.

We divided the whole-brain WM of the normal subjects into 27 structural bundles based on a WM atlas, with 21 WM regions further categorized into left and right hemispheres [34]. In this study, we used SPSS (IBM SPSS Statistics, Version 20.0.0) software for statistical analysis. Multiple testing correction was performed using the Benjamini–Hochberg procedure. This study performs analysis from the perspective of the relative independence of the left and right brain, using independent sample *t*-tests to explore the gradient differences between the left and right brain of subjects based on white matter functional gradients.

### 2.4. Grouping of Corpus Callosum Functional Gradients and Correlation Analysis

In order to better understand how different gradient values in the brain contribute to functional activities, we found the positions of the voxels corresponding to the first 30 and the last 30 of the main gradient values of the CC separately. These voxel positions were then grouped into maximum and minimum value categories to create contrasting gradient value sets. Specifically, in our study, we aimed to explain this using the first functional gradient of the corpus callosum (CC). We first identified the voxel positions corresponding to the 30 largest values in the first gradient of the CC and designated them as the “maximum group”. Similarly, the voxel positions corresponding to the 30 smallest values in the first gradient were referred to as the “minimum group”, allowing for a contrast analysis. Then, we calculated the correlation between the BOLD signals of the corpus callosum and the gray matter regions. Specifically, the BOLD signals of the corpus callosum from all subjects were averaged to obtain the group-level BOLD signal. The time series signals corresponding to the maximum and minimum groups were then aligned within the group-averaged signal. Similarly, the brain gray matter (GM) time signals correspond to the BOLD signals extracted from the 82 structural regions defined by Brodmann partitioning. Pearson’s correlation was then calculated between each gray matter region and the two groups.

## 3. Results

### 3.1. The Functional Gradient in the Hemispheric White Matter

**Figure 2:**
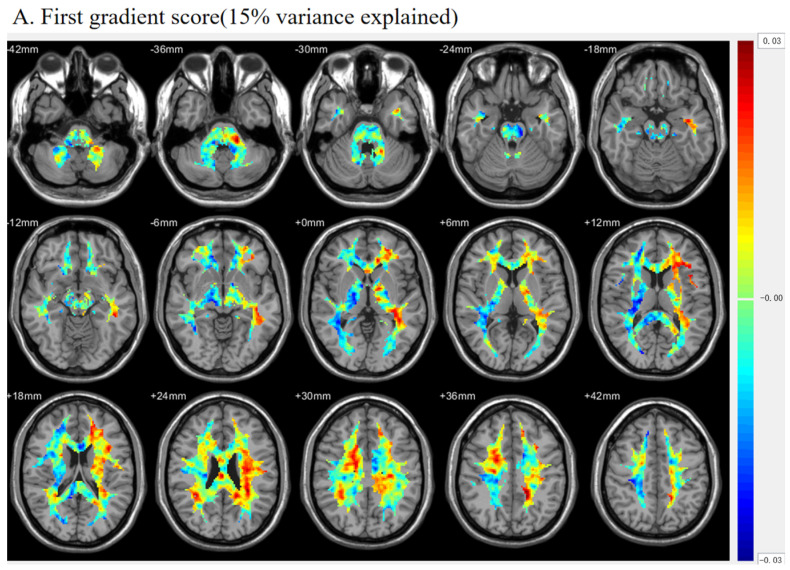

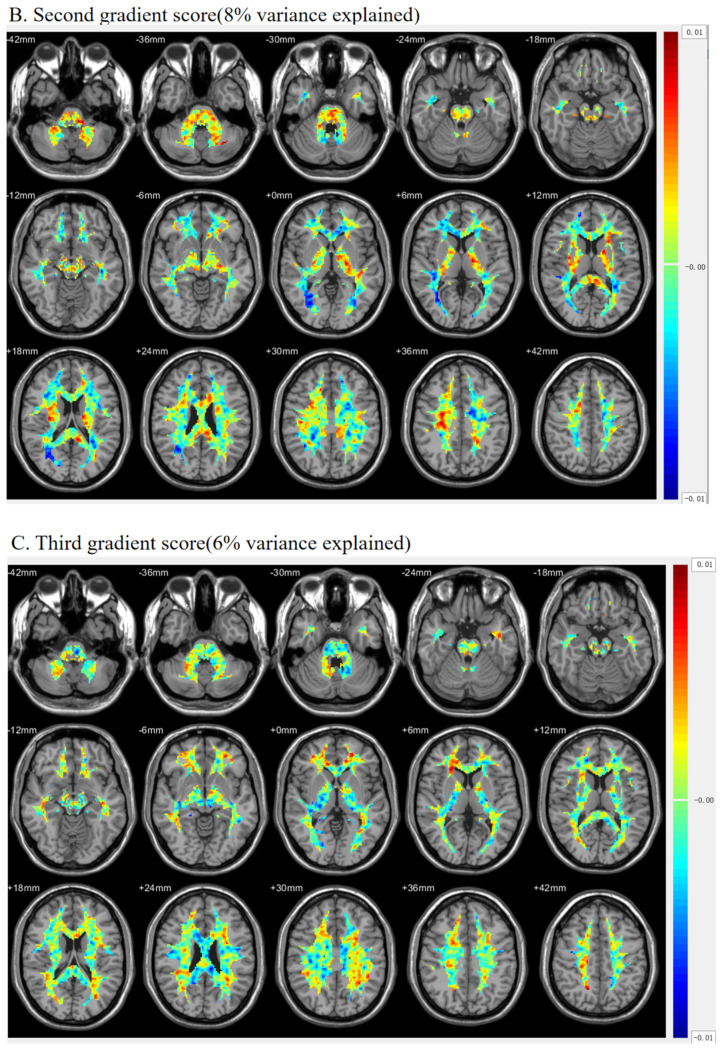
(**A**) The first gradient accounts for 15% of the variance and demonstrates the asymmetric nature of left hemispheric WM and right hemispheric WM. (**B**) The second gradient, accounting for 8% of the variance, demonstrates significant divergence between the anterior and posterior regions of the WM and the central region. (**C**) The third gradient accounts for 6% of the variance and realizes the opposite characteristics of the second gradient.

Different regions on this functional gradient receive distinct scores, reflecting their positions along the spatial axis and indicating their functional distance from each other. In this study, we focused on explaining the first three components of the 29% total variance by mapping the group-level functional gradient (functional gradients averaged across 100 normal subjects) to the WM space to form a gradient visualization map (Figure 2).

**Figure 3:**
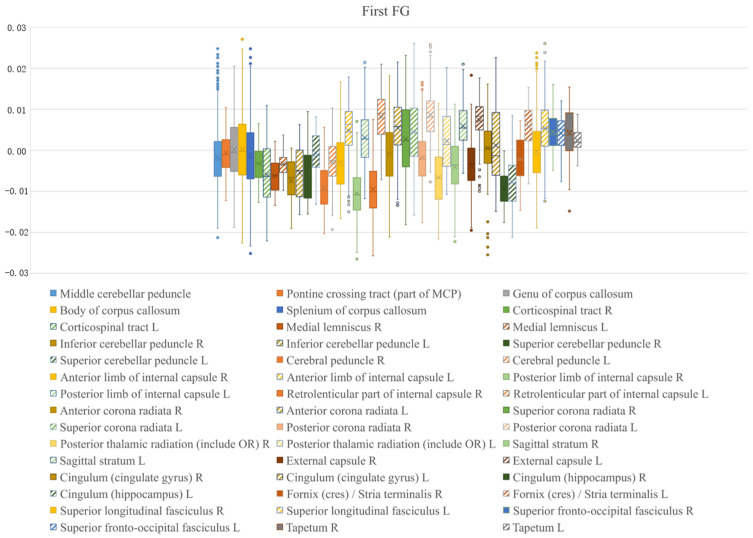
First functional gradient box plot for each WM region.

The principal functional gradient values of all WM bundles (except for the fornix and uncinate fasciculus regions) are presented in box plots (Figure 3). Overall, the range of the principal gradient was significantly greater in the left hemisphere than in the right hemisphere. We investigated the left-right asymmetry and differentiation in the primary functional gradient among the 20 WM structural regions (excluding the uncinate fasciculus region) divided into left and right hemispheres.

We focused on forming gradient visualizations by mapping a group-level functional gradient (functional gradients averaged across 100 normal subjects) into the white matter space. A total of 100 subjects were included (50 males and 50 females).

We validated the results of the independent sample *t*-test by identifying the hemibrain differences and asymmetries in 15 of the 20 WM regions (Table 1), including the corticospinal tract, medial lemniscus, superior cerebellar peduncle, cerebral peduncle, anterior limb of the internal capsule, posterior limb of the internal capsule, retrolenticular part of the internal capsule, anterior corona radiata, superior corona radiata, posterior corona radiata, posterior thalamic radiation, sagittal stratum, external capsule, fornix (cres) and stria terminalis, and superior longitudinal fasciculus. The majority of these regions exhibited higher gradient values in the left hemisphere compared with the right hemisphere (i.e., leftward asymmetry), with only the corticospinal tract region showing higher gradient values in the right hemisphere (AS = all subjects; MS = male subjects; and FS = female subjects).

Subsequently, we grouped the data into male and female groups and performed the same statistical analysis and validation steps separately for each gender. In females, 15 out of 20 WM areas showed hemibrain differences or asymmetries. In males, 15 out of 20 WM areas also showed hemibrain differences or asymmetries. While the numerical differences were equal for both the male and female groups, the specific regions where variances occurred were not the same. Specifically, the corticospinal tract, inferior cerebellar peduncle, superior cerebellar peduncle, and cerebral peduncle regions exhibited different symmetrical properties for different genders (Table 1).

### 3.2. Functional Gradient Distribution of CC

One study found that the estimated mean fractional anisotropy (FA) of the bundle significantly correlated with the fMRI measures of visual cortical activity [36]. Given the significant functional roles of the CC in WM regions, we explored the functional gradients in the CC regions.

**Figure 4:**
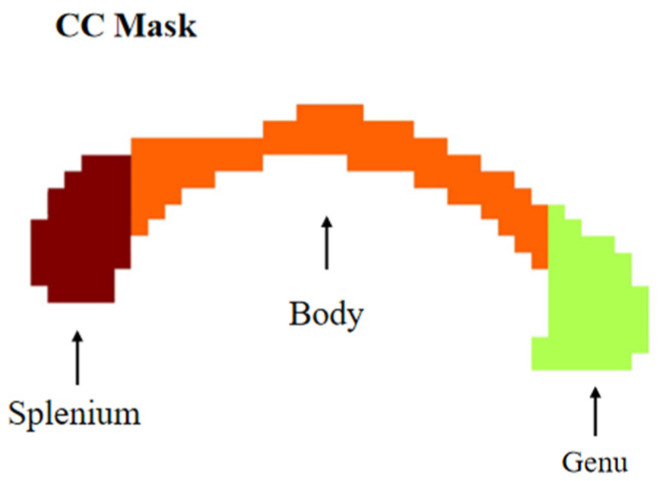
Parcellations of the CC. CC mask from JHU-ICBM-labels-2 mm WM atlas.

We employed a template to capture the precise spatial location of the CC and find the CC functional gradients. The corpus callosum can be divided into seven subregions based on the length of the corpus callosum, including the rostrum, genu, rostral body, anterior midbody, posterior midbody, isthmus, and splenium of the corpus callosum [37]. However, this approach did not define clear boundaries between the different segments. Therefore, we utilized the CC template from the previous section for precise segmentation, including the genu, body, and splenium (Figure 4).

**Figure 5:**
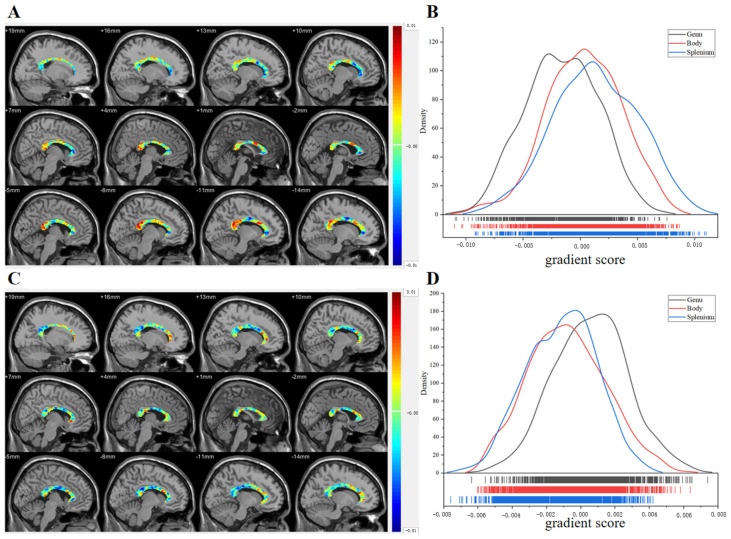
CC functional gradients. (**A**) The second CC functional gradient. (**B**) The distribution of each callosal region in the second gradient. (**C**) The third CC functional gradient. (**D**) The distribution of each callosal region in the third gradient.

Since the previous experiment provided a comprehensive analysis of the asymmetry of the principal functional gradients in the whole-brain white matter, in this experiment, we focused on the second and third gradients of the CC. We mapped the two gradient values onto the CC space (Figure 5A,C), and their distributions were observed and analyzed (Figure 5B,D). When combining the gradient and distribution diagrams, we observed that the higher values in the second gradient (Figure 5A,B) were primarily located in the splenium, with some concentration in the anterior body, lower values were mainly concentrated in the genu, with partial distribution, and intermediate values were mainly concentrated in the body. In the third gradient (Figure 5C,D), higher values were concentrated in the genu, lower values were concentrated in the Splenium and post-body, and intermediate values were mainly concentrated in the anterior body and splenium.

### 3.3. The Relationship Between the WM and GM

**Figure 6:**
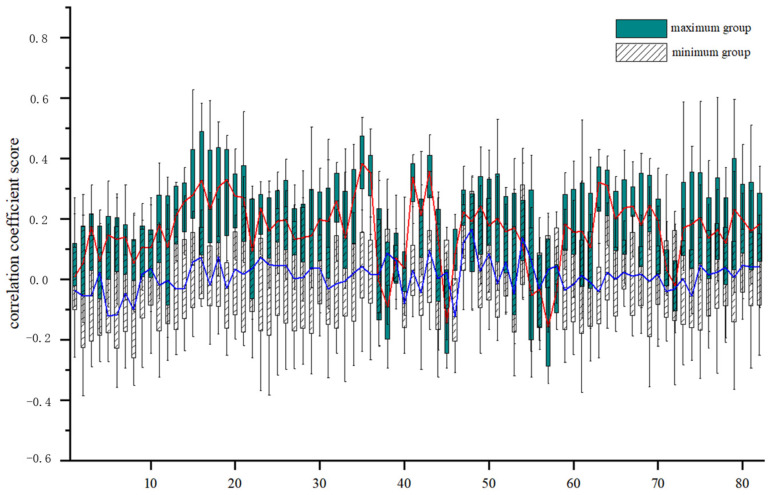
Correlation between high and low gradient WM voxels and 82 GM Brodmann regions (red for maximum group, blue for minimum group).

Specifically, we segmented the brain gray matter into 82 regions using Brodmann partitioning (for details, see Appendix A) and calculated the correlation between each gray matter region and the two subgroups, resulting in two 30 × 82 correlation matrices. After the data were counted, we found that the correlation between the maximum group and GM region was higher than that of the minimum group (Figure 6). However, there were a few isolated instances where the maximal and minimal groups exhibited opposite results in certain GM regions, such as the ventral anterior cingulate cortex, posterior entorhinal cortex (left), perirhinal cortex (on the parahippocampal gyrus), and parahippocampal cortex (on the parahippocampal gyrus). Overall, the disparity in the correlation between the two groups and the gray matter regions was not substantial. Notably, the calculation results of the maximum group were predominantly distributed in the positive half-axis. For the minimum group, although some values extended into the negative half-axis, the majority of the results remained close to zero, indicating a relatively weaker negative correlation.

**Figure 7:**
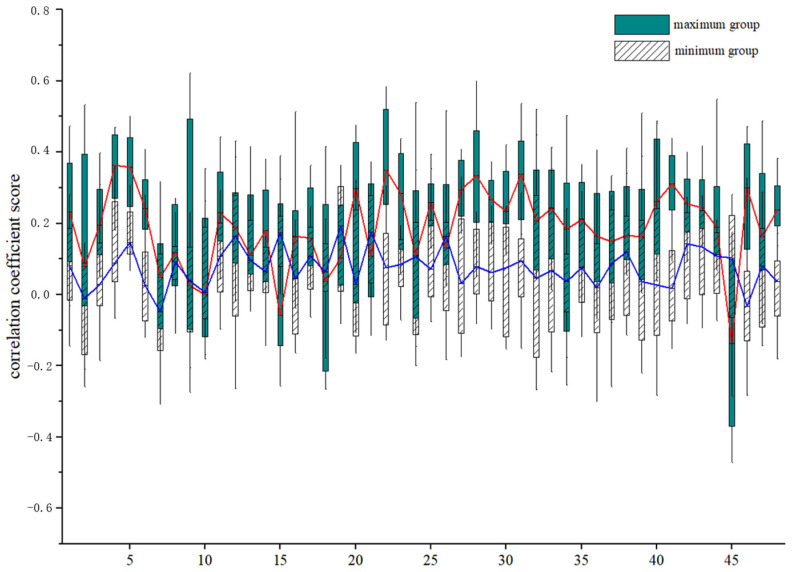
Correlation between high and low gradient WM voxels and 48 WM regions (red for maximum group, blue for minimum group).

Furthermore, by employing the same approach and steps, we also calculated the correlations between 48 WM regions (for details, see Appendix B) and the two subgroups, resulting in two correlation matrices 30 × 48 in size. Intriguingly, the findings of the WM-WM correlations were consistent with the WM-GM correlations mentioned earlier, with the maximum value group exhibiting higher correlation with the WM regions compared with the minimum value group. Additionally, the correlation coefficients of both groups were primarily in the positive half-spectrum, with a smaller gap compared with the previous experiments involving GM (Figure 7). Likewise, a few correlations between the minimal group and the WM structural regions were seen to be higher than the maximal group.

## 4. Discussion

This study used the temporal correlation between voxel-wise time series BOLD signals from WM and GM as the WM FC to calculate the functional gradient of the whole-brain WM and further study the WM of the brain.

### 4.1. Functional Gradient Symmetry Study

Several recent studies have suggested that there are asymmetric sex differences in the human brain’s hemispheres [38]. Liang et al. identified notable differences in the functional gradients of the two brain hemispheres, wherein the left hemisphere in both males and females demonstrated a larger full range of the principal gradient, with males having greater leftward asymmetry [39]. The aforementioned study offers valuable insights into the dissimilarities in the functional hierarchy of the two hemispheres and how they vary. Although for individual subjects, the left brain and the right brain have homology, for a single left brain or right brain, the left brain and the right brain are still independent [40]. However, it is still unclear which particular structures in the human brain’s white matter exhibit functional hemispheric dissimilarities.

The results showed that the principal gradient in the left hemisphere was significantly higher than that in the right hemisphere, which may be related to the fact that the left hemisphere provides more central or indispensable areas for the entire structural network of the brain [41]. This is evidenced by the higher correlations observed between the language regions in the left hemisphere than between their right hemisphere counterparts [42]. For example, the default mode network (DMN) is unevenly distributed functionally and structurally between the hemispheres and is predominantly left-brained [43]. The asymmetry in hemispheric functional gradients we observed may also be related to the previously reported tendency of left hemispheric regions to interact intrahemispherically and the right hemispheric regions to interact more bilaterally, with greater information flow from the right to the left hemisphere, stronger FC from the right to the left prefrontal cortex, and higher correlations between the left hemispheric language regions than between their right hemispheric cognates [44,45].

The results of our experiment suggest that sex has a significant impact on the hemispheric functional gradient, which is consistent with the well-established notion that brain asymmetry is associated with sex [46,47]. Notably, the brain’s structure and function differ significantly between genders. For example, Peper et al. highlighted the crucial link between sex hormones and FC in the brain [48]. Furthermore, research has demonstrated that females generally perform better in language processing, while males tend to have superior visuospatial abilities [49]. This may be due to the larger volume of temporal and parietal regions (around the lateral fissure) involved in language processing in females and the noted larger volume of the parietal cortex associated with visuospatial function in males. These findings suggest that there may be gender-dependent processing patterns within each hemisphere, which could partly explain respective male and female advantages in distinct behavioral and cognitive functions. The hemispheric functional gradient and its asymmetry provided new insights and ideas for understanding human brain asymmetries and re-emphasized the important influence of gender.

### 4.2. The Function Gradient Value Distribution in the CC

The cortical areas of the two hemispheres of the brain interact through the CC [40]. Additionally, the CC plays a crucial role in the interhemispheric transmission of homotopic functions such as sensory and motor functions and higher cognition [50]. Another study found that using surgery to sever the CC connection can severely affect interhemispheric FC in humans [51], consistent with the above findings. Thus, it is considered to be the key to the collaborative functional integration between the cerebral hemispheres and of particular importance in humans.

Even though the CC plays a critical part in brain functions, current segmentation methods rely mainly on its cytoarchitecture, geometry, and topological projection. There is little attention given to the functional organization of the brain regions it connects. Therefore, callosal regions delineated based on atlases do not necessarily coincide with their functional roles in the brain. Friedrich et al. complemented this by using a functional gradient associated with the functional hierarchy [52]. Consequently, they found that the apex of the principal gradient of the CC extended from the genu to the posterior body with low connectivity variability. In this context, the term “connective voxels in the callosal midsection” refers to specific regions within the corpus callosum where voxels exhibit strong functional connectivity with other brain regions, particularly across the midsection. These voxels are identified based on their involvement in facilitating communication between the two cerebral hemispheres. Additionally, the number of these connective voxels decreased gradually toward both ends of the gradient.

To illustrate the CC functional gradients, we mapped them back to the CC space and plotted the second and third functional gradients (Figure 5). Upon analyzing the distribution of the two gradient values in the CC, we observed that the posterior body had a predominantly lower distribution of either the second or third gradient values. Previous research has shown that fibers in the posterior midbody project to lower-level processing locations [39]. The genu is linked to higher-level associative regions (such as the prefrontal and orbitofrontal cortices). The spatial distribution of the anatomical functional organization of the CC and the third gradient map of the CC (Figure 5B) assumed in these studies was consistent. However, the second gradient plot does not align with these assumptions, and both the second and third gradient values in the splenium and genu regions had opposite distributions. Previous studies have demonstrated that cortical regions with strong structural connectivity often exhibit high functional connectivity. However, in regions where structural connectivity is weak but functional connectivity remains high, the direct relationship between structure and function tends to be less evident [53,54]. These findings suggest that the structure–FC relationship is not one-to-one. Therefore, the third gradient of the CC more accurately reflects its anatomical structure, whereas the second gradient may disclose additional functional levels of the CC. Future research can explore this perspective further.

### 4.3. Correlation Study

Initially, the WM of the brain was considered a crucial conduit for conveying information between various GM subdivisions. Therefore, there exists a clear correlation between the functional activity of the WM and GM. To achieve a deeper understanding of this relationship, we employed the primary functional gradient of the CC to elucidate this. We calculated that the correlation between the BOLD signal of the voxel time series corresponding to the maximal group in the main gradient of the CC and the 82 Brodmann subdivisions of the GM was basically positive and greater than that of the minimal group (Figure 6). This outcome may indicate that voxels which correspond to regions with higher gradient values are relatively more active or important in the functional activities of the brain’s gray matter. Nevertheless, we found that the correlation between the minimal group and a few gray matter regions was higher than that of the maximal group, namely the ventral anterior cingulate cortex, posterior entorhinal cortex (left), perirhinal cortex (on the parahippocampal gyrus), and parahippocampal cortex (on the parahippocampal gyrus). Notably, three of these regions—the posterior entorhinal cortex (left), perirhinal cortex (on the parahippocampal gyrus), and parahippocampal cortex (on the parahippocampal gyrus)—are involved in neural activities related to the hippocampus, including olfactory, hippocampal processing, and hippocampal associative functions. The hippocampus, which is situated in the medial temporal lobe, is a complex structure which establishes extensive connections with the anterior and posterior cortices, thereby enabling the hippocampus to synchronize a diverse array of network activities. The functional connection between the white matter and hippocampus can be a new research direction in the future, and the functional brain activity of the white matter can be explored in depth.

Employing the same strategy, we ascertained the correlations between the two groups and 48 WM regions and established stronger correlations between the same maximal group and WM (Figure 7). These results may serve as supporting evidence that voxels which correspond to higher gradients tend to be more active. However, we observed that, akin to the correlation experiments between gray matter regions, a few white matter regions displayed a stronger correlation with the minimal group than the maximal group. We suggest that this may be due to their involvement in specific neural activity, as distinct neural activities activate different WM regions.

### 4.4. Limitations

This study relied on a specific dataset (i.e., the HCP dataset), which may have limited the generalizability and external validity of the findings. While this dataset provides high-quality resting-state fMRI data, future studies should validate this approach using other independent samples or datasets to confirm its broad applicability.

The method presented in this study has not been fully assessed for test-retest reliability or external validation. To ensure the robustness and reproducibility of the proposed approach, future research should conduct test-retest analyses to assess consistency across different time points or experimental conditions. Additionally, external validation using different datasets is crucial to enhance the generalizability and stability of the method.

This study used the diffusion map embedding algorithm to estimate the functional gradients in white matter. While this method performed well with the current dataset, it requires further validation across different brain regions and sample populations. Additionally, certain parameters within the method (e.g., the α value) may have influenced the results. Future research should explore the impact of these parameters and optimize them for more accurate analysis.

## 5. Conclusions

We provide a complete functional gradient map of the brain’s WM, providing additional ideas for studying the structure and function of WM. We explored the asymmetry observed in the functional hierarchy of the brain, evaluated the distribution of functional gradient values of the brain’s largest WM bundles (CC), and utilized the functional gradient to examine the relationship between WM and GM. Future studies may use whole-brain WM functional gradients to delve deeper into the deeper relationships between WM and GM FC.

## Figures and Tables

**Table 1:** First functional gradient box plot for each WM region.

WM Regions	AS	MS	FS	WM Regions	AS	MS	FS
Corticospinal tract	<0.001	0.26	<0.001	Posterior corona radiata	<0.001	<0.001	<0.001
Medical lemniscus	0.001	<0.001	0.009	Posterior thalamic radiation	<0.001	<0.001	<0.001
Inferior cerebellar peduncle	0.08	0.01	0.82	Sagittal stratum	<0.001	<0.001	<0.001
Superior cerebellar peduncle	<0.001	0.68	<0.001	External capsule	<0.001	<0.001	<0.001
Cerebral peduncle	<0.001	<0.001	0.51	Cingulum (cingulate gyrus)	0.56	0.19	0.29
Anterior limb of internal capsule	<0.001	<0.001	<0.001	Cingulum (hippocampus)	0.19	0.85	0.07
Posterior limb of internal capsule	<0.001	<0.001	<0.001	Fornix(cres) and stria terminalis	<0.001	<0.001	<0.001
Retrolenticular part of internal capsule	<0.001	<0.001	<0.001	Superior longtitudinal fasciculus	<0.001	<0.001	<0.001
Anterior corona radiata	<0.001	<0.001	<0.001	Superior fronto-occipital fasciculus	0.69	<0.001	<0.001
Superior corona radiata	<0.001	0.001	<0.001	Tapetum	0.18	0.51	0.15

## Data Availability

The data used in this study are publicly available from the Human Connectome Project (HCP) dataset. Access to the dataset can be requested through the official HCP website (https://www.humanconnectome.org/) under their terms and conditions. Further inquiries can be directed to the corresponding authors.

## Appendix A

**Brodmann ROIs**			
1	Primary somatosensory cortex1 L	42	Retrosubicular area R
2	Primary somatosensory cortex2 L	43	Pars orbitalis, part of the inferior frontal gyrus R
3	Primary somatosensory cortex3 L	44	Dorsolateral prefrontal cortex R
4	Primary motor cortex L	45	Pars triangularis, part of the IFG and part of Broca R
5	Somatosensory association cortex L	46	Pars opercularis, part of the IFG and part of Broca R
6	Premotor and supplementary motor cortex L	47	Primary gustatory cortex R
7	Visuomotor coordination L	48	Auditory cortex2 R
8	Includes frontal eye fields L	49	Auditory cortex1 R
9	Dorsolateral prefrontal cortex L	50	Supramarginal gyrus, may be part of Wernicke R
10	Anterior prefrontal cortex L	51	Angular gyrus, may be part of Wernicke R
11	Orbitofrontal area L	52	Temporopolar area R
12	Primary visual cortex (V1) L	53	Fusiform gyrus R
13	Secondary visual cortex (V2) L	54	Ectorhinal area, now part of the perirhinal cortex R
14	Associative visual cortex (V3, V4, V5) L	55	Perirhinal cortex (in the rhinal sulcus) R
15	Inferior temporal gyrus L	56	Dorsal entorhinal cortex (parahippocampal gyrus) R
16	Middle temporal gyrus L	57	Dorsal anterior cingulate cortex R
17	Superior temporal gyrus L	58	Part of cingulate cortex R
18	Ventral posterior cingulate cortex L	59	Retrosplenial cingulate cortex R
19	Ventral anterior cingulate cortex L	60	Ventral entorhinal cortex R
20	Subgenual area L	61	Piriform cortex R
21	Ectosplenial portion of the retrosplenial region L	62	Ectosplenial portion of the retrosplenial region R
22	Piriform cortex L	63	Subgenual area R
23	Ventral entorhinal cortex L	64	Ventral anterior cingulate cortex. R
24	Retrosplenial cingulate cortex L	65	Ventral posterior cingulate cortex R
25	Part of cingulate cortex L	66	Superior temporal gyrus R
26	Dorsal anterior cingulate cortex L	67	Middle temporal gyrus R
27	Dorsal entorhinal cortex (parahippocampal gyrus) L	68	Inferior temporal gyrus R
28	Perirhinal cortex L	69	Associative visual cortex (V3, V4, V5) R
29	Ectorhinal area, now part of the perirhinal cortex L	70	Secondary visual cortex (V2) R
30	Fusiform gyrus L	71	Primary visual cortex (V1) R
31	Temporopolar area L	72	Orbitofrontal area R
32	Angular gyrus, may be part of Wernicke L	73	Anterior prefrontal cortex R
33	Supramarginal gyrus, may be part of Wernicke L	74	Dorsolateral prefrontal cortex R
34	Auditory cortex1 L	75	Includes frontal eye fields R
35	Auditory cortex2 L	76	Visuomotor coordination R
36	Primary gustatory cortex L	77	Premotor and supplementary motor cortex R
37	Pars opercularis, part of IFG and part of Broca L	78	Somatosensory association cortex R
38	Pars triangularis, part of IFG and part of Broca L	79	Primary motor cortex R
39	Dorsolateral prefrontal cortex L	80	Primary somatosensory cortex3 R
40	Pars orbitalis, part of the inferior frontal gyrus L	81	Primary somatosensory cortex2 R
41	Retrosubicular area L	82	Primary somatosensory cortex1 R

## Appendix B

**WM ROIs**			
1	Corticospinal tract L	25	Body of corpus callosum
2	Medial lemniscus L	26	Splenium of corpus callosum
3	Inferior cerebellar peduncle L	27	Fornix (column and body of fornix)
4	Superior cerebellar peduncle L	28	Tapetum R
5	Cerebral peduncle L	29	Uncinate fasciculus R
6	Anterior limb of internal capsule L	30	Superior fronto-occipital fasciculus R
7	Posterior limb of internal capsule L	31	Superior longitudinal fasciculus R
8	Retrolenticular part of internal capsule L	32	Fornix (cres) and stria terminalis R
9	Anterior corona radiata L	33	Cingulum (hippocampus) R
10	Superior corona radiata L	34	Cingulum (cingulate gyrus) R
11	Posterior corona radiata L	35	External capsule R
12	Posterior thalamic radiation (includes OR) L	36	Sagittal stratum R
13	Sagittal stratum L	37	Posterior thalamic radiation (includes OR) R
14	External capsule L	38	Posterior corona radiata R
15	Cingulum (cingulate gyrus) L	39	Superior corona radiata R
16	Cingulum (hippocampus) L	40	Anterior corona radiata R
17	Fornix (cres) and stria terminalis L	41	Retrolenticular part of internal capsule R
18	Superior longitudinal fasciculus L	42	Posterior limb of internal capsule R
19	Superior fronto-occipital fasciculus L	43	Anterior limb of internal capsule R
20	Uncinate fasciculus L	44	Cerebral peduncle R
21	Tapetum L	45	Superior cerebellar peduncle R
22	Middle cerebellar peduncle	46	Inferior cerebellar peduncle R
23	Pontine crossing tract (part of MCP)	47	Medial lemniscus R
24	Genu of corpus callosum	48	Corticospinal tract R
